# Development, dissemination and community response towards the first community notice regarding misrepresented illicit anabolic‐androgenic steroids in circulation in Australia

**DOI:** 10.1111/dar.14015

**Published:** 2025-02-10

**Authors:** Timothy Piatkowski, Isabelle Volpe, Rita Brien, Ross Coomber, Monica Barratt, Emma Kill, Geoff Davey, Cameron Francis, Sarah Cresswell, Alan White, Madeline Harding, Karen Blakey, Steph Reeve, Brooke Walters, Jason Ferris, Cheneal Puljević

**Affiliations:** ^1^ School of Applied Psychology Griffith University Mount Gravatt Australia; ^2^ Griffith Centre for Mental Health Griffith University Brisbane Australia; ^3^ Queensland Injectors Voice for Advocacy and Action Sunshine Coast Australia; ^4^ The Loop Australia Brisbane Australia; ^5^ Social Equity Research Centre and Digital Ethnography Research Centre RMIT University Melbourne Australia; ^6^ Drug Policy Modelling Program, Social Policy Research Centre UNSW Sydney Sydney Australia; ^7^ Department of Sociology, Social Policy and Criminology, Faculty of Humanities and Social Sciences University of Liverpool Liverpool UK; ^8^ National Drug and Alcohol Research Centre UNSW Sydney Sydney Australia; ^9^ Queensland Injectors Health Network Brisbane Australia; ^10^ School of Environment and Science Griffith University Brisbane Australia; ^11^ Centre for Health Services Research The University of Queensland Brisbane Australia; ^12^ School of Public Health The University of Queensland Brisbane Australia

**Keywords:** anabolic‐androgenic steroids, drug checking, harm reduction, image and performance‐enhancing drugs, oxandrolone

## Abstract

**Introduction:**

Drug alerts aimed at both people who use drugs and health workers help to prevent acute harms from unpredictable illicit drug markets and by equipping health workers to handle unusual drug events and share vital information with service users. However, there has never been an alert produced for anabolic‐androgenic steroids (AAS), an important class of illicit drugs. We report on the development, implementation and community receptivity of the first‐ever AAS community drug alert.

**Methods:**

Based on findings from samples collected during the first month of an AAS checking trial conducted by drug checking service CheQpoint, we identified contamination in two oxandrolone samples, which prompted issuing of the first‐ever AAS community notice. Drawing on digital ethnographic techniques, we collected and analysed social media comments on the notice to assess AAS community perceptions and the broader impact of this harm reduction initiative.

**Results:**

The Instagram post by CheQpoint reached 1376 users, with 3429 impressions and 87 interactions. Community feedback indicated receptivity to the notice, with several people in the community recognising the prevalence of AAS adulteration. Responses highlighted the need for more thorough testing and indication of sample content, given the perception of a growing number of new people using AAS.

**Discussion and Conclusions:**

This study, the first to describe a community notice for illicit market AAS, reveals a strong demand for harm reduction interventions. We call for the urgent expansion of drug‐checking services to provision for AAS and, thus, provide equitable health support to address systemic gaps for this group.

## 1 INTRODUCTION

People who use anabolic‐androgenic steroids (AAS) typically acquire their AAS from an unregulated and illicit market due to legal constraints, prescription requirements, limited accessibility, and social stigma [1]. Previous research has highlighted the risks associated with the lack of regulation in the black market, leading to uncertainty about product quality [2]. Specifically, clandestine laboratories manufacturing these drugs have been linked to counterfeit substances with incorrect dosages, undisclosed ingredients and contamination risks, with approximately two‐thirds of such drugs being of unknown origin and content [3]. This can serve to potentiate the already increased health risks of AAS use, which can result in serious health issues, including organ damage, hypertension, psychiatric disorders and increased risk of sudden cardiac death [4, 5]. As a result of the surging illicit AAS market [6, 7], it follows that the risks of receiving counterfeit AAS and, therefore, being exposed to health risks, is a matter of concern to the individual using AAS.

Drug‐checking services offer chemical analysis of substances as well as the delivery of health information for people who use these substances, with the goal of reducing risks associated with consumption of drugs and promoting safer drug use among people who use drugs [8]. However, drug‐checking services that test AAS remain scarce due to significant logistical and technical challenges, including the need for costly off‐site testing, validated methods, reference standards and specialised staff [2]. In Australia, this gap in health and harm reduction is evident, as CheQpoint, Queensland's first drug checking service with sites in Brisbane and Gold Coast currently hosts the world's first and only AAS checking trial. Despite this progress, there remains a significant gap in alert systems tailored specifically to AAS, limiting opportunities to address unexpected risks within this group.

Drug alerts warn of unexpected drug components and are shared via social media and other channels to prevent harm by equipping communities and health workers to respond to adverse events [9, 10]. However, alerts for AAS are rare. Localised systems like WEDINOS in the UK have previously tested and distributed alerts for similar enhancement drugs such as dinitrophenol, with people using these drugs reporting concerns regarding contamination [11]. However, the scope and distribution of alerts regarding these substances were limited, with few issued and primarily disseminated through specialised channels, and the UK's testing of enhancement drugs ceased in 2014 [12]. Here, we report on the development, implementation and community receptivity of what we believe to be the first broadly disseminated AAS drug alert about unexpected products detected in AAS samples tested.

## 2 METHODS

The alert described in this study relates to AAS samples collected as part of an AAS checking trial conducted by drug checking service, CheQpoint, located in Queensland, Australia. Table 1 shows Queensland's available drug‐checking services and their processes.

**TABLE 1:** Processes in place for testing and alerts in Queensland, Australia.

Queensland service/program	Description	Implementation	Target audience
CheQpoint Fixed‐Site Drug Checking Service	Free, confidential drug‐checking service to identify substances' chemical composition and reduce harm. Includes fixed‐site testing, mobile services and community alerts	Launched in April 2024 with fixed sites in Brisbane and Gold Coast and mobile testing at festivals and events	General public, particularly people who use drugs
On‐Site Festival Drug Testing (operated by CheQpoint and Pill Testing Australia)	Real‐time drug analysis at festivals and events, providing immediate feedback on substance content and purity	Implemented at events such as Rabbits Eat Lettuce Festival and Schoolies celebrations	Event attendees, primarily youth and young adults
Community Alert System	Disseminates warnings about dangerous substances detected in the community by drug checking and health/forensic testing services, using social media and other communication channels	Alerts issued based on emerging threats	People who use drugs, health workers and the community

**FIGURE 1:**
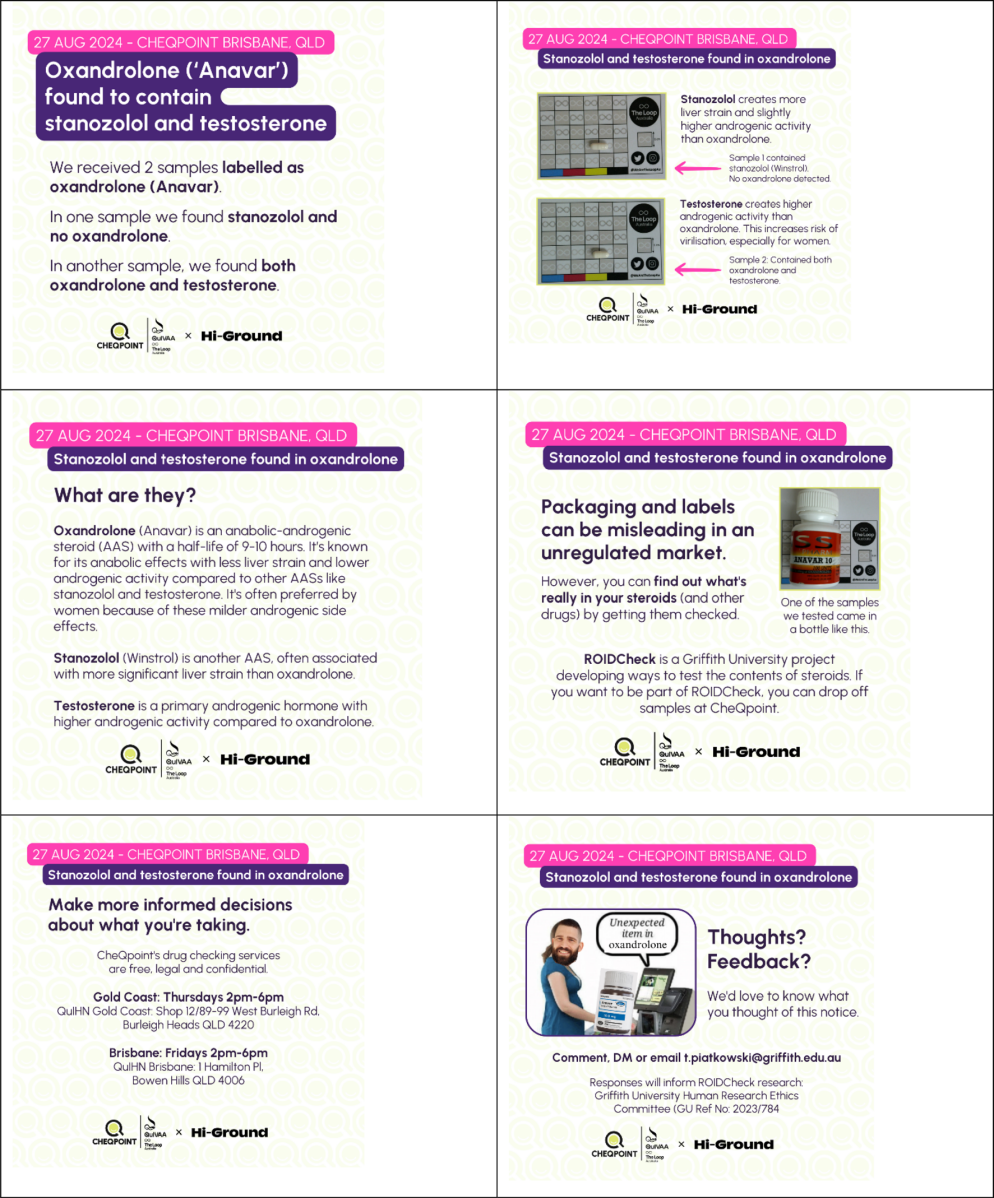
Oxandrolone community notice.

CheQpoint's AAS trial operated within this context and, in doing so, identified contamination in two oxandrolone samples [13]; one sample contained no oxandrolone and was found to be stanozolol, while the other had oxandrolone mixed with testosterone. Recognising the risks of oxandrolone, which has been reported to be frequently substituted in the illicit AAS market [14], we produced a community notice based on these findings to reduce harm (see Figure 1). This community notice was crafted in close collaborative partnership between the first and second authors, with broader input from the research team. The lead author is an established peer‐researcher with specialised expertise in AAS and other enhancement drugs. Drawing on insights from CheQpoint, the research team's collective knowledge and the relevant literature, as well as the lead author's lived‐living experience, the notice was developed to address critical issues in this domain.

Following the notice, we invited community feedback and conducted an analysis of the responses to assess its impact. Ethical approval was granted from the Griffith University Human Research Ethics Committee (Approval: 2023/784). Recognising that the community notice would be shared online, we informed the AAS community via social media about its release and associated research. We explicitly stated on the social media posts that all responses—whether public comments or private messages—would be collected and analysed. To address ethical considerations, all data were de‐identified during analysis and private messages were accessible only to the research team.

To contextualise the community responses to our notification, we employed a digital ethnographic approach [15]. These types of approaches are commonplace when exploring drug use and community responses on social media [16, 17]. In this study, we monitored Instagram comments and direct messages, as well as emails from the community in response to our notice. This methodology draws from established digital ethnographic practices [18] to capture the diverse ways in which participants engaged with our content. Through a process of open and inductive coding [19], we initially identified broad content categories, such as engagement type, perceptions of harm and community expectations. These codes were subsequently refined into more specific subcategories, including types of feedback (e.g., positive or neutral responses), consumer experiences with adulterated substances, and the perceived value of testing and notices.

## 3 RESULTS

### 3.1 Engagement analyses

The post was published as an Instagram post at 16:21 on 27 August 2024 and was not boosted (i.e. promoted with paid advertising). The post was published on the Instagram account of CheQpoint (@cheqpoint.qld), which at the time had 704 followers.

Within a 1‐week timeframe, the Instagram post was seen 3429 times, by 1376 unique users and had 87 total interactions (including 70 likes, 3 comments, 0 shares and 12 saves). The lead author received 10 direct messages (@timpiatkowski) and 4 emails about the post, each representing a unique individual response.

### 3.2 Perceptions of harm and community feedback

The general tone in the comments and direct messages indicated that the community appreciated the harm reduction initiative.

‘Great job guys’ [Instagram comment #1]

‘We would love to have this kind of service over here (Canada)’ [Instagram comment #2]

This generally positive attitude was also reflected in the direct messages received:

‘In regard to the earlier post; that kind of social media content does provide valuable feedback to the community. Real results based on real samples from the community shows that care and caution needs to be taken in using any form of unregulated compound.’ [Instagram message #3]

### 3.3 Experience with adulteration and counterfeit substances

Several people expressed that they were not surprised by the adulteration of oxandrolone, suggesting that the expectation of compromised substances was widespread. For instance, one person commented:

‘Surprise surprise (not surprised).’ [Instagram comment #3]

This was echoed in messages such as:

‘That was one of mine. Not surprised by the results for that one either.’ [Instagram message #2]

Several additional direct messages reflected a broader recognition of the pervasive issues of underdosing and counterfeiting in the illicit AAS market, highlighting an accepted reality among people who use AAS.

‘I quickly realised that most stuff was underdosed [less active ingredient than listed] or counterfeit. We would love to have the capacity to test our stuff.’ [Instagram message #4]

### 3.4 Impact on harm reduction practices

The community notice had a direct practical impact, particularly for health workers. One respondent, who was a current health worker, shared an example of encouraging a person to bring their substance for testing:

‘I saw a girl in the NSP [needle service provider] today was getting sharps for her partner. We got talking, she is taking Anavar [oxandrolone]. I told her about this [community notice], and she said she felt her last delivery of Anavar ‘felt different’ during and after training. I encouraged her to bring it in tonight to CheQpoint and get it tested.’ [Instagram message #8]

This quote illustrates how health workers can facilitate harm reduction by sharing information directly with people who use AAS, leading to increased awareness and encouraging proactive actions, such as testing substances. This quote also demonstrates that people who use AAS noted that they could detect the effects of adulterated substances, using alert information alongside their own bodily awareness to interpret these unfamiliar experiences.

### 3.5 Awareness of the need for drug‐checking services

Feedback via email highlighted the perceived need for more drug‐checking services, particularly in the context of new consumers:

‘[…] a huge amount of new people are getting on performance enhancers without engaging in the level of research that they ‘should’ be doing before taking the plunge. Thats where this drug check information is highly valuable […] to stand up against the amount of advertisement by PED [performance enhancing drug] users on these social media platforms. Specifically drug testing: exposing what this new market demographic are consuming may not be what they think it is. And that drug checking is important, especially for new users.’ [Email message #1]

The feedback highlighted a critical issue in the current AAS market: the influx of younger initiates who lack adequate information and research before using AAS. The respondent emphasised that community notices provided essential counterbalance to the extensive marketing and glorification of these substances on social media, particularly for less experienced people.

### 3.6 Constructive feedback on testing visibility and outreach

There was some constructive feedback provided regarding the current scale and visibility of AAS checking efforts.

‘The level of testing and broadcasting of this type of testing and results do not stand up against the volume of how many new users there are. The only negative of the current testing [AAS testing trial] and broadcasting of results is there is not enough of it being done, and the broadcasting needs to be on a bigger platform more available to the public.’ [Email message #3]

A few respondents acknowledged the logistical challenges of drug checking for AAS but underscored the insufficient extent and outreach of current testing and result dissemination. They advocated for increased testing capacity and broader, more accessible public communication to better address the needs of the expanding user base.

## 4 DISCUSSION

This study is the first known Australian effort to create a community notice for illicit market AAS, marking a significant contribution to harm reduction. By developing and implementing this notice, we provide a foundation for similar initiatives and a documented example to guide future efforts. The positive community response highlights a clear demand for such harm reduction and health promotion interventions. This is important given that current harm reduction services are often ill‐equipped to meet the needs of this population [20, 21], which is further compounded by stigma and the invisibility of AAS consumers within conventional health and public policy frameworks [1, 22]. These structural limitations not only hinder health‐seeking behaviours but also often leave many people reliant on informal networks for information and support, which may perpetuate misinformation and unsafe practices [23]. Expanding drug‐checking services for AAS represents an opportunity to address these gaps in two key ways. First, drug checking directly empowers individuals with accurate, actionable information about the substances they use, helping them to make safer choices. Second, it serves as an engagement tool, providing a critical entry point for connecting people who use PIEDs with broader harm reduction and health services.

Interestingly, participants in this study used the term ‘underdosed’ to describe the issue of low active ingredient content in AAS. This contrasts with conventional illicit drug markets, where ‘adulteration’ is the more typical term for substances being compromised [24]. This distinction highlights the unique language of the AAS community [25], suggesting that while terminology may differ, the underlying concern about substance integrity and safety remains central.

Despite considerable community interest through private messages and emails, public interaction via shares and comments was low, perhaps reflecting the broader systemic challenges faced by people who use AAS [1], including stigma [22]. This may also point to the larger, yet largely hidden population of AAS consumers who are not engaged with services, a reality that has been highlighted in a recent study from the UK, where the prevalence of AAS use was found to be significantly higher than previously estimated [26]. Many AAS consumers remain outside of formal support systems, making it crucial to use digital platforms and online fora to engage this population with harm reduction messages. Ultimately, these findings emphasise the importance of building infrastructure that directly addresses the needs of people who use AAS, ensuring more inclusive and effective harm reduction outcomes.

## AUTHOR CONTRIBUTIONS

Timothy Piatkowski: Conceptualisation, Funding Acquisition, Methodology, Formal Analysis, Investigation, Data Curation, Writing – Original Draft, Review and Editing. Isabelle Volpe: Formal Analysis, Investigation, Data Curation, Writing – Review and Editing. Rita Brien: Investigation, Data Curation, Writing – Review and Editing. Ross Coomber: Formal Analysis, Writing – Review and Editing. Monica Barratt: Writing – Original Draft, Writing – Review and Editing. Emma Kill: Funding Acquisition, Writing – Review and Editing. Geoff Davey: Writing – Review and Editing. Cameron Francis: Investigation, Writing – Review and Editing. Sarah Cresswell: Investigation, Writing – Review and Editing. Alan White: Investigation, Writing – Review and Editing. Madeline Harding: Investigation, Writing – Review and Editing. Karen Blakey: Investigation, Formal Analysis, Writing – Review and Editing. Steph Reeve: Writing – Review and Editing. Brooke Walters: Funding Acquisition, Writing – Review and Editing. Jason Ferris: Formal Analysis, Writing – Review and Editing. Cheneal Puljević: Writing – Review and Editing, Supervision.

## CONFLICT OF INTEREST STATEMENT

Drs Piatkowski, Puljević, Barratt, Prof Ferris, Emma Kill, Alan White, Madeline Harding and Karen Blakey are volunteer members of The Loop Australia, which is a national organisation for drug checking and drug checking research. Isabelle Volpe and Rita Brien are communications officers of The Loop Australia and Cameron Francis is the CEO of The Loop Australia. Geoff Davey is the CEO of Queensland Injectors Health Network. Emma Kill is the CEO of Queensland Injectors Voice for Advocacy and Action, and Dr Piatkowski is the Vice President of the organisation.

## Data Availability

The data that support the findings of this study are available from the corresponding author upon reasonable request.
